# DNA-induced synthesis of biomimetic enzyme for sensitive detection of superoxide anions released from live cell†

**DOI:** 10.1039/c7ra12962a

**Published:** 2018-04-03

**Authors:** Ailing Ding, Bin Wang, Xiaoqing Ma, Jianglin Diao, Jiushang Zheng, Jiucun Chen, Changming Li

**Affiliations:** Institute for Clean Energy & Advanced Materials, Faculty of Materials and Energy, Southwest University Chongqing 400715 P. R. China bwang@swu.edu.cn ecmli@swu.edu.cn

## Abstract

In this work, we successfully fabricate a rapid, sensitive sensor for the detection of superoxide anions O_2_˙^−^ based on graphene/DNA/Mn_3_(PO_4_)_2_ biomimetic enzyme. In the design, graphene is served as excellent carrier to improve the catalysis of Mn_3_(PO_4_)_2_ nanoparticles; and DNA adsorbed on graphene acts as medium to assist the growth of Mn_3_(PO_4_)_2_ on graphene. The fabricated graphene/DNA/Mn_3_(PO_4_)_2_ composites exhibit excellently electrochemical activity, significantly decrease the response time and increase the sensitivity of the sensor towards O_2_˙^−^. The successful detection of O_2_˙^−^ released from cancer cell demonstrated its potential applications in biology and medicine.

## Abstract 2

In this work, we successfully fabricate a rapid, sensitive sensor for the detection of superoxide anions O_2_˙^−^ based on graphene/DNA/Mn_3_(PO_4_)_2_ biomimetic enzyme.

## Graphical abstract

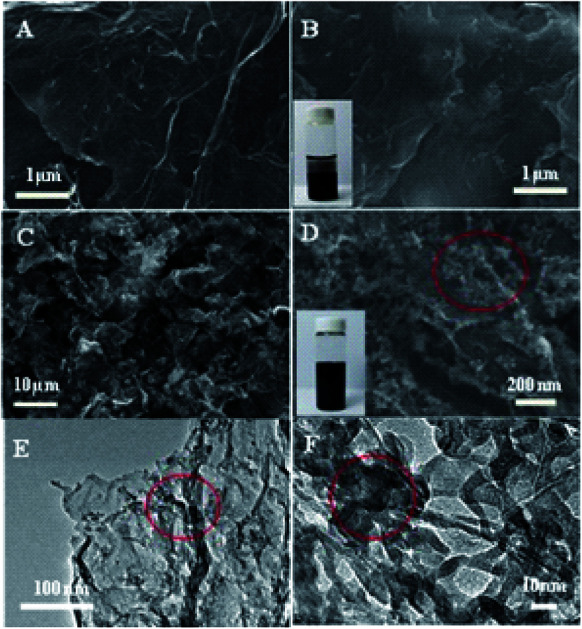

## Introduction

Superoxide anions (O_2_˙^−^) are the primary type of reactive oxygen species (ROS). Under normal conditions, O_2_˙^−^ is highly reactive and unstable and its metabolism is a rapid and spontaneous process. Overproduction of O_2_˙^−^ can cause various disease, such as aging, asthma, ulcer disease, cancer, atherosclerosis, neurodegenerative diseases and other diseases.^1–4^ Thus the detection of O_2_˙^−^ is very important in various biological systems. Many methods have been developed for the detection of O_2_˙^−^ including chemiluminescent, spectrophotometric, fluorometric, and electrochemical technique, *etc.*^5–8^ Among all these techniques, electrochemical method receives most attention due to its advantages of real-time assay, high sensitivity and selectivity. And most of the reported electrochemical methods for O_2_˙^−^ detection are based on enzyme catalysts such as cytochrome *c* and superoxide dismutase (SOD).^2,9–11^ Though enzyme improves the assay performance of sensors, but its high cost and poor long-term stability increase difficulty of direct monitoring of O_2_˙^−^ in biological samples. Therefore, it is a serious challenge for us to establish a fast, reliable, and sensitive non-enzyme approach for O_2_˙^−^ monitoring in physiological and pathological processes.

As early as 1982, manganese was reported possessing an effective catalytic effect *in vivo* protection against superoxide toxicity.^12^ In recent years, further study found that manganese phosphate (Mn_3_(PO_4_)_2_) has the ability to catalyze the dismutation of O_2_˙^−^ compared with free Mn^2+^ ion that only stoichiometrically reacts with O_2_˙^−^.^13^ Therefore Mn_3_(PO_4_)_2_ is usually selected as a substitute for enzyme to detect O_2_˙^−^.^14^ Mao *et al.* synthesize SiO_2_-Mn_3_(PO_4_)_2_ nanoparticles derived from phytic acid and utilize for O_2_˙^−^ detection.^15^ Lan and co-workers deposit Mn_3_(PO_4_)_2_ nanoparticles on carbon nanomaterials directly for the fabrication of O_2_˙^−^ sensor.^16^ These works verify the catalysis of Mn-superoxide dismutase mimics towards O_2_˙^−^. But the synthesis and property of nanoparticles need further study to improve the catalytic performance of sensor. In this work, we synthesize Mn_3_(PO_4_)_2_ nanoparticles by DNA induction, which results in even dispersion and excellent catalytic activity towards O_2_˙^−^ disproportionation reaction.

Graphene is an increasingly important nanomaterials due to its excellent electronic conductivity, good stability, and promising catalytic performance in sensing.^17–20^ However, the two dimensional structure is easy to aggregate due to the single-atom-thickness, and hydrophobic aromatic structure. To solve this problem, we functionalize graphene with deoxyribonucleic acid (DNA) which can be adsorbed on graphene through π–π stacking and don't destroy the intact structure of the carbon materials.^20^ Under the induction of ssDNA, Mn_3_(PO_4_)_2_ nanoparticles was evenly deposited on the surface of graphene. The obtained graphene/DNA/Mn_3_(PO_4_)_2_ nanocomposites displayed significant biomimetic enzyme activity, rapid and sensitive response towards O_2_˙^−^. This approach holds a great promise for broad applications in biomedical research and clinical test.

## Experimental section

### Chemicals and materials

MCF-7 was bought from Cell bank of the representative culture preservation committee of the Chinese Academy of Sciences, China. Manganese sulfate (MnSO_4_), potassium phosphate tribasic (K_3_PO_4_) and Nafion were purchased from East Sichuan Chemical Industry (Group) Co., Ltd. (Chongqing, China) and used as received. Graphene was obtained from Sinocarbon Materials Technology Co., Ltd., China. Zymosan A (Zym, from *Saccharomyces cerevisiae*) SOD, DNA (low molecular weight extracted from salmon sperm), and potassium superoxide were purchased from Sigma-Aldrich and used without further purification. The O_2_˙^−^ solutions were prepared by dissolving KO_2_ in PBS solution (pH 7.0, N_2_ saturated). The concentration of O_2_˙^−^ was determined by the reduction of ferri cytochrome *c* spectrophotometrically.^21^ All the other solutions were prepared using deionized water (18 MΩ cm) and were degassed with high purity nitrogen before experiments. All electrochemical experiments were carried out at room temperature.

### Apparatus and instrumentations

Scanning electron microscopy (SEM) images were taken by JSM-6510LV, Japan. Field emission scanning electron microscopy (FESEM) images were taken by JSM-7800F, Japan. X-Ray diffraction (XRD) measurements were performed on a Shimadzu diffractometer (XRD-7000, Tokyo, Japan) operating in reflection mode with Cu Kα radiation at a step size of 0.06 per second. The nanosheets of graphene/DNA/Mn_3_(PO_4_)_2_ were characterized by transmission electron microscopy (TEM, JEM-2100F, Japan). Fourier transform infrared spectroscopy (FTIR) spectra were determined using a Thermo Nicolet 6700 FTIR spectrometer. Electrochemical performances were characterized using a CHI 660 system (Shanghai Chenhua, China). Graphene/DNA/Mn_3_(PO_4_)_2_ modified glassy carbon electrode (graphene/DNA/Mn_3_(PO_4_)_2_/GCE) served as working electrodes, platinum sheet as counter electrode and Ag/AgCl (in 3 M KCl) acted as reference electrode. All O_2_˙^−^ measurements were performed in 10 mL 0.01 M PBS solution (pH = 7.4).

### Synthesis of graphene/DNA/Mn_3_(PO_4_)_2_ nanosheets

Graphene/DNA/Mn_3_(PO_4_)_2_ nanosheets were prepared by the previously reported method.^22^ Thirty milligrams of DNA was dissolved in 30 mL de-ionized water under stirring, after which the solution was annealed at 95 °C for 15 min to produce single-stranded DNA (ssDNA). Then the obtained ssDNA was mixed with 15 mL of 1 mg mL^−1^ graphene by mildly sonicated at 4 °C for 2 h, thereafter filtration and washing were performed to remove excess ssDNA. Subsequently, the mixture was dispersed into 0.1 M MnSO_4_ solution, then 0.1 M K_3_PO_4_ solution were dropwisely added under stirring and kept at room temperature for 30 min. Then graphene/DNA/Mn_3_(PO_4_)_2_ nanosheets were obtained by centrifugation at 8000 rpm for 10 minutes and washing with deionized water for 3 times.

Glassy carbon electrode (GCE, *d* = 3 mm) was polished with alumina slurry to a smooth and bright surface, washed by sonication for 30 s and dried under nitrogen. Then 5 μL of the obtained graphene/DNA/Mn_3_(PO_4_)_2_ suspension was dropped on the electrode surface and dried at room temperature. Finally, 5% Nafion solution was coated to stabilize the fabricated electrode.

### *In situ* detection of O_2_˙^−^ released form living cells

MCF-7 cells were cultured in a humidified incubator (95% air with 5% CO_2_) at 37 °C. The cells were cultured in Dulbecco's Modified Eagle's Medium (DMEM) (Cellgro, USA) supplemented with 1 mol L^−1^ glutamine, 50 U mL^−1^ penicillin/streptomycin and 10% heat inactivated fetal bovine serum.^14^ For the detection of O_2_˙^−^ released from living cell, the incubation solution was removed and washed with PBS solution (pH 7.4) for three times. Before electrochemical measurement, zymosan (Zym) was added to motivate cells generation of O_2_˙^−^. Amperometric response was recorded by CHI-660B electrochemical station at applied potential of 700 mV (*versus* Hg/Hg_2_Cl_2_).

## Results and discussion

### Design of the modified graphene nanosheets

**Scheme 1:**
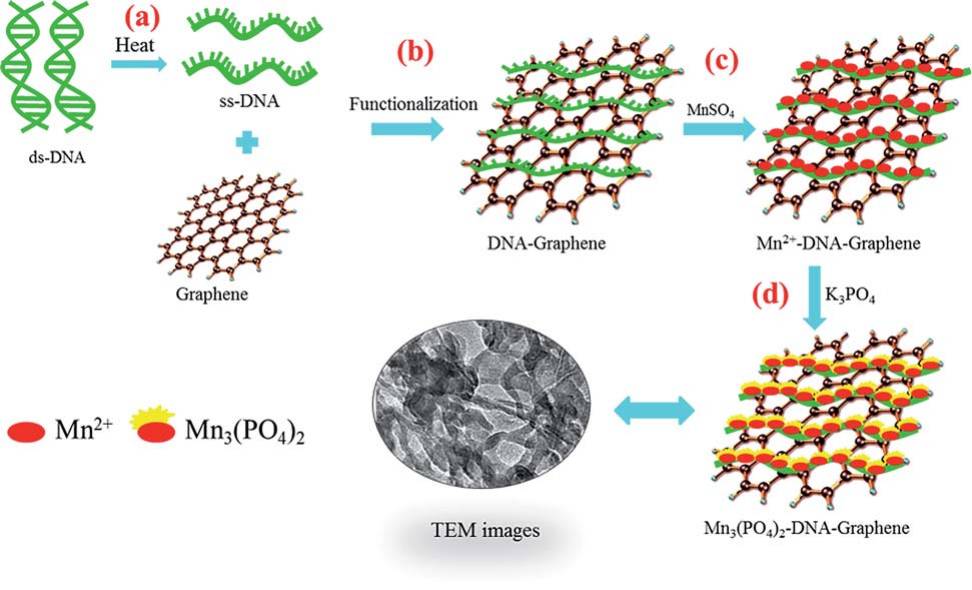
Schematic illustration of the preparation procedure for the graphene/DNA/Mn_3_(PO_4_)_2_.

Scheme 1 illustrates the synthesis of graphene/DNA/Mn_3_(PO_4_)_2_ enzyme mimics by growth of Mn_3_(PO_4_)_2_ on graphene under the induction of DNA. As we all know, single-strand DNA (ssDNA) can be adsorbed on graphene by π–π interaction.^23,24^ Here ssDNA was prepared by annealing double-strand DNA (dsDNA) and applied for graphene modification. In the presence of Mn^2+^, the divalent cations accumulate along the ssDNA backbone by the electrostatic interaction, which facilitate the formation of Mn_3_(PO_4_)_2_ crystal nucleus on graphene and growth of Mn_3_(PO_4_)_2_ nanoparticles upon the addition of negatively charged PO_4_^3−^ groups.

### Characterization of graphene/DNA/Mn_3_(PO_4_)_2_ nanocomposites

**Fig. 1:**
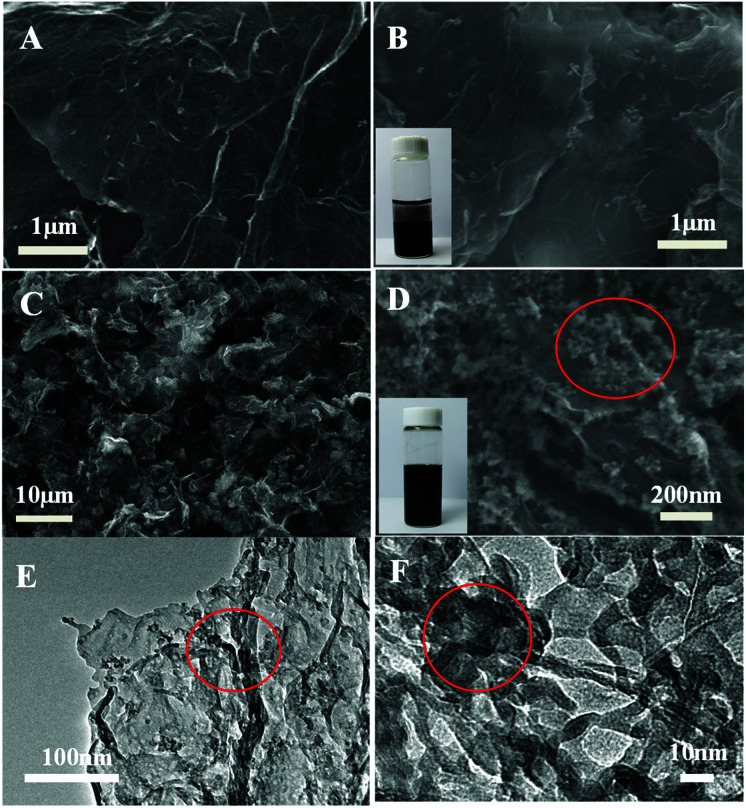
(A) SEM images of pure graphene, (B): SEM images of graphene/Mn_3_(PO_4_)_2_ formed without DNA template, (C) and (D): SEM images of graphene/Mn_3_(PO_4_)_2_ formed with DNA template, (E) and (F): TEM images of graphene/Mn_3_(PO_4_)_2_ formed with DNA template. (The Mn_3_(PO_4_)_2_ nanoparticles was labeled with red circle).

The morphology of the as-prepared graphene/DNA/Mn_3_(PO_4_)_2_ nanosheets is characterized with scanning electron microscope (SEM). Fig. 1A and B shows the SEM images of graphene before and after adding MnSO_4_ and K_3_PO_4_, typical wrinkle of graphene can be clearly observed and no evident Mn_3_(PO_4_)_2_ nanoparticles is found in Fig. 1B, suggesting that it is difficult to form Mn_3_(PO_4_)_2_ nanoparticles on graphene surface without DNA. However, when ssDNA is modified on graphene (Fig. 1C and D), even-distributed Mn_3_(PO_4_)_2_ nanoparticles can be evidently observed on the surface of graphene. The phenomena reveal the crucial role of ssDNA on the growth of Mn_3_(PO_4_)_2_ on graphene. The TEM images in Fig. 1E and F display different morphology of Mn_3_(PO_4_)_2_ nanoparticles due to the overlapping of particles. In order to verify the results, energy disperse spectrometer (EDS) was performed to explore chemical composition of the materials. The EDS results in Fig. S2† indicate that the major constituent elements of the wirelike nanostructure conclude O (68.7 at%), P (12.1 at%) and Mn (19.2 at%), and the atomic ratio of Mn and P is close to 3 : 2, suggesting the existence of Mn_3_(PO_4_)_2_ nanoparticles. The results further confirm the formation of well-defined thin-layer structure of the nanocomposites of Mn_3_(PO_4_)_2_ nanoparticles on graphene surface.

**Fig. 2:**
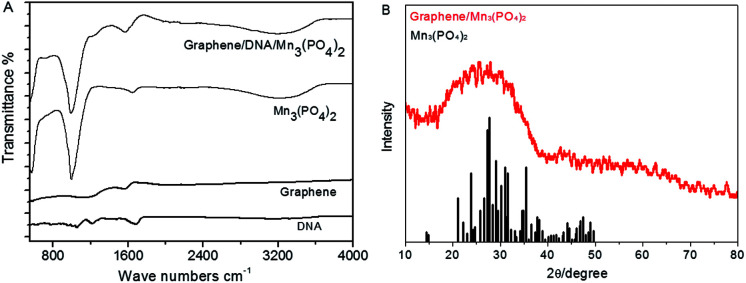
(A): FTIR image of different materials; (B): X-ray diffraction pattern of graphene/DNA/Mn_3_(PO_4_)_2_ nanosheets (red line) and the standard values of Mn_3_(PO_4_)_2_ (black line).

Then FTIR spectra are determined to further verify the formation of graphene/DNA/Mn_3_(PO_4_)_2_ nanosheets. As shown in Fig. 2A, absorption peak at 1568.6 cm^−1^ can be assigned to the aliphatic carboxylic acid salts, the peaks at 996.0 cm^−1^ and 3196.3 cm^−1^ were attributed to inorganic phosphates. The results suggest that DNA and Mn_3_(PO_4_)_2_ have been successfully modified on the graphene. In addition, the X-ray diffraction pattern (XRD) of graphene/Mn_3_(PO_4_)_2_ in Fig. 2B shows that the diffraction peak of the nanomaterials is at around 28.2°, which is consistent with the results of Mn_3_(PO_4_)_2_. The results further confirm that Mn_3_(PO_4_)_2_ nanoparticles are successfully modified on the surface of graphene. Then the zeta potential at various stages of the synthesis were measured to further characterize the formation of the nanocomposites. The results in Fig. S1† indicate that the surface charge of graphene is about −0.203 mV, which decreases to −32.5 mV as DNA was adsorbed on its surface due to the negative charge of DNA backbone. When Mn_3_(PO_4_)_2_ was deposited, the zeta potential increase to −15.6 mV, indicating the formation of graphene/DNA/Mn_3_(PO_4_)_2_ nanocomposites.

### Electrochemical properties of graphene/DNA/Mn_3_(PO_4_)_2_ nanosheets

**Fig. 3:**
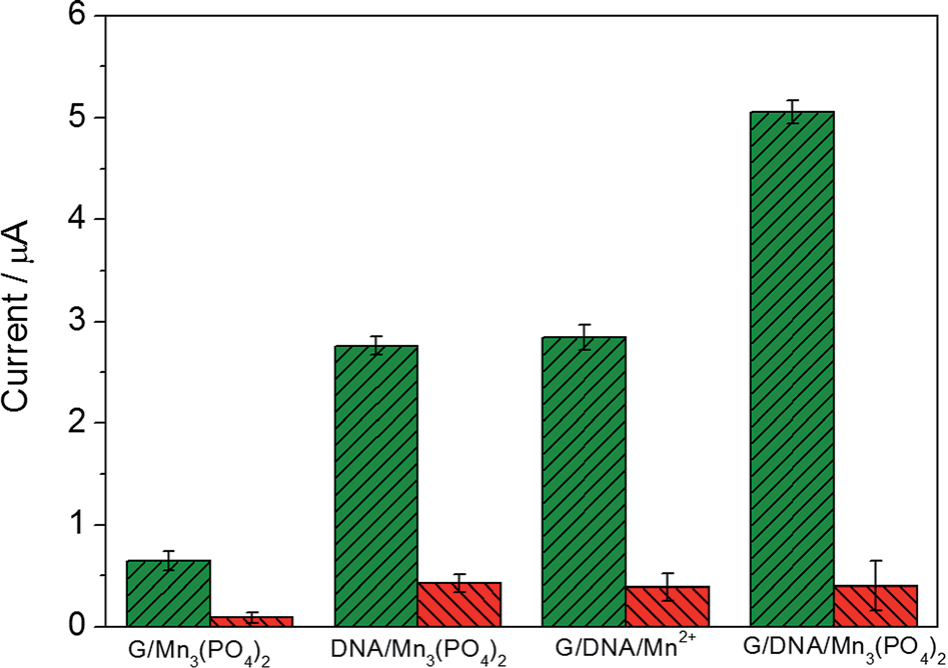
Current response of graphene/Mn_3_(PO_4_)_2_/GCE, Mn_3_(PO_4_)_2_/DNA/GCE, graphene/DNA/Mn^2+^/GCE and graphene/DNA/Mn_3_(PO_4_)_2_/GCE towards O_2_˙^−^ (green column) and the mixture of O_2_˙^−^ and SOD (red column). The error bar was obtained from three measurements.

Fig. 3 exhibits the electrochemical response of different materials in the absence and presence of 1.0 μM O_2_˙^−^ and the mixture of O_2_˙^−^ and SOD (red column) in PBS. When graphene/DNA/Mn_3_(PO_4_)_2_ nanosheets were modified on GCE, the obtained modified electrode displayed evident redox peaks around 0.7 V and 0.5 V in the absence of O_2_˙^−^ (as shown in Fig. S3†), which can be attributed to the electrochemical transformation between Mn^2+^ and Mn^3+^ species. However, no evident current response is observed when the mixture of O_2_˙^−^ and SOD is added, suggesting the current response attributed to the catalysis of the graphene/DNA/Mn_3_(PO_4_)_2_ nanosheets towards O_2_˙^−^. These results verify the biomimetic enzyme activity of graphene/DNA/Mn_3_(PO_4_)_2_ to catalyze the dismutation of O_2_˙^−^.^25^

In order to further study the electrochemical performance of graphene/DNA/Mn_3_(PO_4_)_2_ nanosheets, graphene/Mn_3_(PO_4_)_2_ were synthesized by direct depositing Mn_3_(PO_4_)_2_ nanoparticles on graphene in the absence of DNA, DNA/Mn_3_(PO_4_)_2_ nanoparticles were by prepared depositing Mn_3_(PO_4_)_2_ on dsDNA template and graphene/DNA/Mn^2+^ was synthesized by adsorbing DNA on graphene and subsequent depositing Mn^2+^ on DNA. Electrochemical performance of these materials were explored with electrodes modified with the above three nanomaterials (Fig. 3A–C). As shown in Fig. 3A, the electrode modified with graphene/Mn_3_(PO_4_)_2_ (graphene/Mn_3_(PO_4_)_2_/GCE) possesses excellent electrical conductivity but the current response towards O_2_˙^−^ is weak, verifying the good conductivity of graphene and poor modification of Mn_3_(PO_4_)_2_ (Fig. 3A). Similarly, the electrodes modified with Mn_3_(PO_4_)_2_/DNA and graphene/DNA/Mn^2+^ display evident current response to O_2_˙^−^, but the current response is lower than that on graphene/DNA/Mn_3_(PO_4_)_2_/GCE (as shown in Fig. 3B and C). The phenomena further prove that DNA plays important roles in the synthesis of graphene/DNA/Mn_3_(PO_4_)_2_ nanosheets. In addition, in comparison with the Mn_3_(PO_4_)_2_/DNA/GCE (Fig. 3B), we can see that the graphene/DNA/Mn_3_(PO_4_)_2_/GCE (Fig. 3D) displays stronger current signal due to the excellent conductivity of graphene.

The reason for DNA facilitating the growth of Mn_3_(PO_4_)_2_ nanoparticles on graphene can be ascribed to two factors. First, the π–π interaction between DNA and graphene induces ordered assembly of DNA on the surface of graphene, which is a critical factor to the synthesis of Mn_3_(PO_4_)_2_ nanoparticles. Next, Mn^2+^ is adsorbed on DNA backbone to assist the nucleation of Mn_3_(PO_4_)_2_ nanoparticles. In addition, in comparison with other conventional method, the DNA induced synthesis is easier to control the morphology and dispersion of products by adjusting temperature, pH, or DNA concentration.

### Electrochemical response of O_2_˙^−^ at the present electrode

**Fig. 4:**
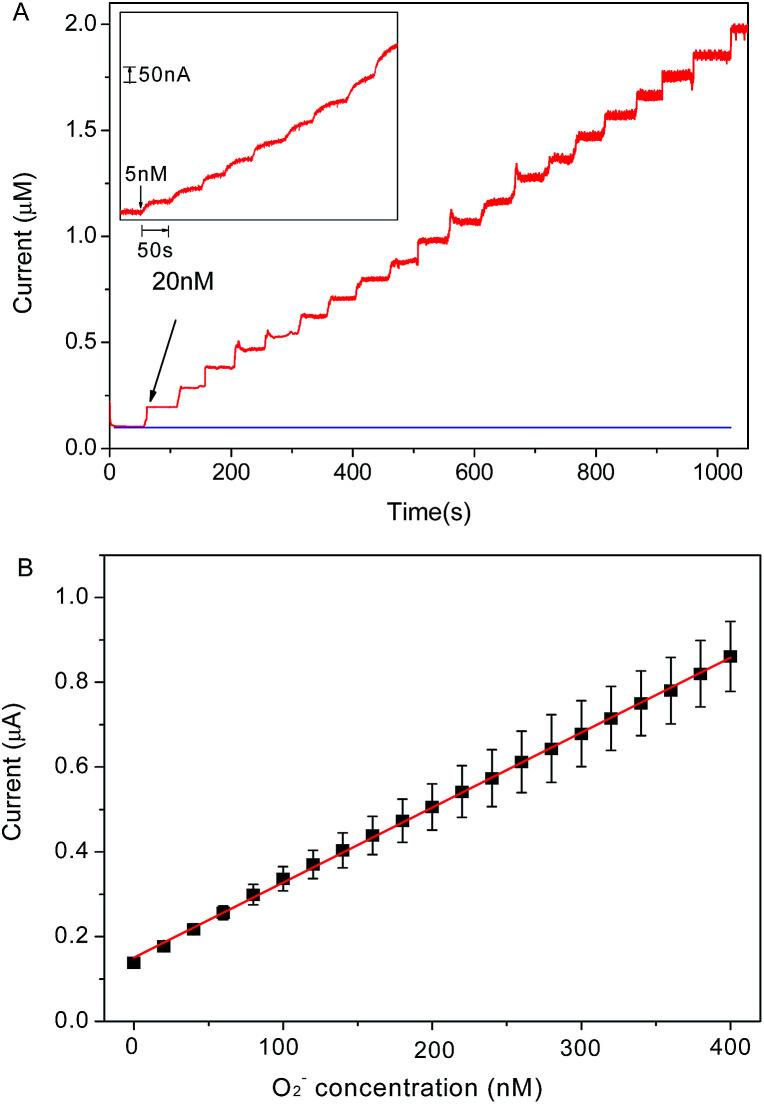
(A): Typical amperometric responses of graphene/DNA/Mn_3_(PO_4_)_2_/GCE to successive additions of 20 nM O_2_˙^−^ and 5 nM O_2_˙^−^ (inset) at applied potentials of 700 mV *versus* Ag/AgCl in 10 mM PBS (pH 7.4); (B): linear plot for O_2_˙^−^ detection.

Chronoamperometry is utilized to investigate the electrochemical response of graphene/DNA/Mn_3_(PO_4_)_2_/GCE towards O_2_˙^−^ in 10 mM PBS (pH = 7.4). Fig. 4A shows the stepwise current response of the present electrode with successive increasing O_2_˙^−^ concentration from 5 nM to 400 nM. From the results in Fig. 4B we can see that the prepared sensor displays significant current response to the O_2_˙^−^ concentrations in the range of 5 nM to 400 nM with a regression equation expressed as *I* (μA) = 0.00354 *c* (nM) + 0.1504 (*R*^2^ = 0.999). The detection limit of the assay is calculated to be about 1.67 nM (S/N = 3) with a sensitivity of 3.54 μA μM^−1^, which is much more superior to most of previous reports.^14,23,26–28^ The response time of the electrode to O_2_˙^−^ is as short as 5 s, which is an advantage for the detection of O_2_˙^−^ due to its short lifetime. These results demonstrate that the present sensor may meet the requirement of O_2_˙^−^ detection in normal physiological conditions.^29^

### The selectivity and stability of the present electrode

**Fig. 5:**
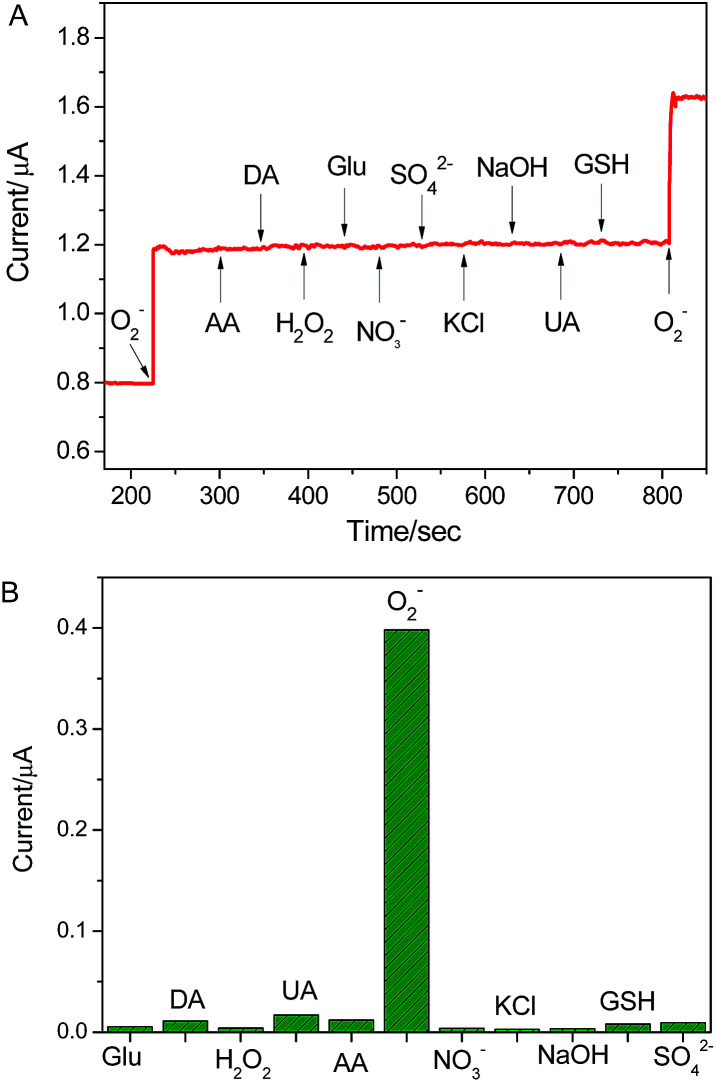
The selectivity of graphene/DNA/Mn_3_(PO_4_)_2_/GCE for the assaying of O_2_˙^−^ against the different interfering species. The response obtained upon the addition of 10 μM interferes and 100 nM O_2_˙^−^.

The selectivity experience was carried out in the presence of various interfering species, such as K^+^, Na^+^, Cl^−^, NO_3_^−^, SO_4_^2−^, hydrogen peroxide (H_2_O_2_), glucose (Glu), glutathione (GSH) uric acid (UA) and ascorbic acid (AA). Among these interferences, H_2_O_2_, UA and AA possess good electrochemical activity and perplex the detection of O_2_˙^−^ due to their wide existence in biological systems. As shown in Fig. 5, in comparison to the electrochemical response of the as-present sensor to 100 nM O_2_˙^−^, the presence of 10 μM K^+^, Na^+^, Cl^−^, SO_4_^2−^, NO_3_^−^, Glu. GSH, AA, UA and H_2_O_2_ did not cause any noticeable current response. The result suggests that the proposed sensor possesses excellent specificity to O_2_˙^−^ detection in biological systems. In addition, the reproducibility and stability of the electrode were explored. The electrochemical response of the sensor was performed using five different electrodes fabricated at the same time, the standard derivations of current response was lower than 5.2%. When the electrode was stored at 4 °C for one month, the current response towards O_2_˙^−^ decrease 6.8% of original value (as shown in Fig. S4†). These results indicate that the excellent reproducibility and stability of the sensor.

### Detection of O_2_˙^−^ released from cancer cell

**Fig. 6:**
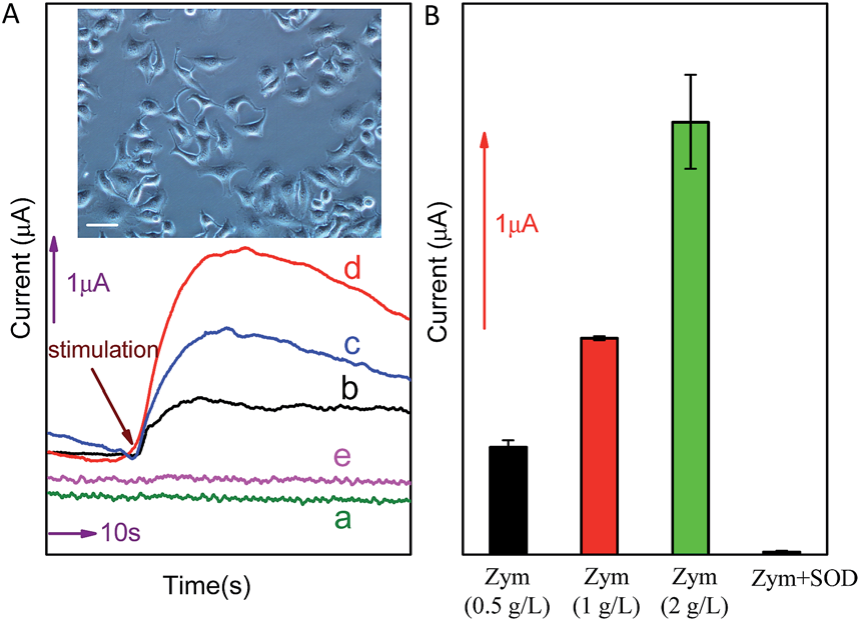
(A): Electrochemical response of graphene/DNA/Mn_3_(PO_4_)_2_/GCE to O_2_˙^−^ released by living MCF-7 cells (with the density of 105 mL^−1^) which stimulated by 0 g L^−1^ (a), 0.5 g L^−1^ (b), 1 g L^−1^ (c), 2 g L^−1^ (d) Zym and 1 g L^−1^ Zym + 250 U mL^−1^ SOD (e) in PBS. Inset, MCF-7 cell used in the determination (scale bar: 50 μm). (B): The histogram of (A).

In order to further investigate the potential application of the method in biological systems, the detection of O_2_˙^−^ released from MCF-7 cells was performed at ambient temperature. As shown in Fig. 6, upon the addition of different concentration (0.5 g L^−1^, 1.0 g L^−1^ or 2.0 g L^−1^) of zymosan (Zym, a drug was used to stimulate living cells to release O_2_˙^−^), a significant current response was observed (corresponding to O_2_˙^−^ oxidation). According to the calibration curve in Fig. 5B, the concentration of O_2_˙^−^ released from MCF-7 cells is calculated to be around 156.38 nM, 315.71 nM and 631.81 nM, respectively (the concentration of O_2_˙^−^ released by per cell and total cells are calculated and listed on Table 1). The results indicate that current response of O_2_˙^−^ released form cancer cell under Zym stimulation is a concentration-dependent behavior. In addition, the results of control experiments (curve *d* and *e* shown in Fig. 6) indicate that no current was obtained upon the addition of either Zym or SOD-Zym mixture, confirming that the current responses were attributed to the drug-induced O_2_˙^−^ release from cells.

**Table 1:** Current response and the concentration of O_2_˙^−^ released by MCF-7 cells (with the density of 10^5^ mL^−1^) under stimulation of different concentration of Zym

Stimulation	Response current (μA)	Concentration of O_2_˙^−^ released by cells (nM)	O_2_˙^−^ released by per cell (pM)
Zym 0.5 g L^−1^	0.704	156.38	0.782
Zym 1 g L^−1^	1.268	315.71	1.579
Zym 2 g L^−1^	2.387	631.81	3.159

## Conclusions

A novel O_2_˙^−^ biomimetic enzyme sensor based on graphene/DNA/Mn_3_(PO_4_)_2_ nanosheets is successfully developed. The synthesized nanosheets display strong electrocatalytic activity towards O_2_˙^−^ with high sensitivity, excellent selectivity and fast current response. The successful determination of O_2_˙^−^ released from cancer cells demonstrates the great potential for application in biological system. This work provides a new method for the fabrication of biomimetic enzyme sensor and a great promise for biosensing and biomedical application.

## Conflicts of interest

There are no conflicts to declare

## Supplementary Material

RA-008-C7RA12962A-s001
