# Supplementary material for: DNA-induced synthesis of biomimetic enzyme for sensitive detection of superoxide anions released from live cell
Source: RSC Adv. 2018 Apr 3;8(22):12354–9. doi: 10.1039/c7ra12962a (PMC9079267; doi:10.1039/c7ra12962a)

Electronic Supplementary Material (ESI) for RSC Advances.
This journal is © The Royal Society of Chemistry 2018

Supporting information

DNA-induced Synthesis of Biomimetic Enzyme for Sensitive Detection of Superoxide Anions released from live cell

Ailing Ding, Bin Wang*, Xiaoqing Ma, Jianglin Diao, Jiushang Zheng, Jiucun Chen and Changming Li*

Institute for Clean Energy & Advaced Materials, Faculty of Materials and Energy, Southwest University, Chongqing 400715, P.R. China.

E-mail: bwang@swu.edu.cn, ecmli@swu.edu.cn

Fig S1. Zeta potential of graphene, graphene/DNA and graphene/DNA/$Mn_3(PO_4)_2$.

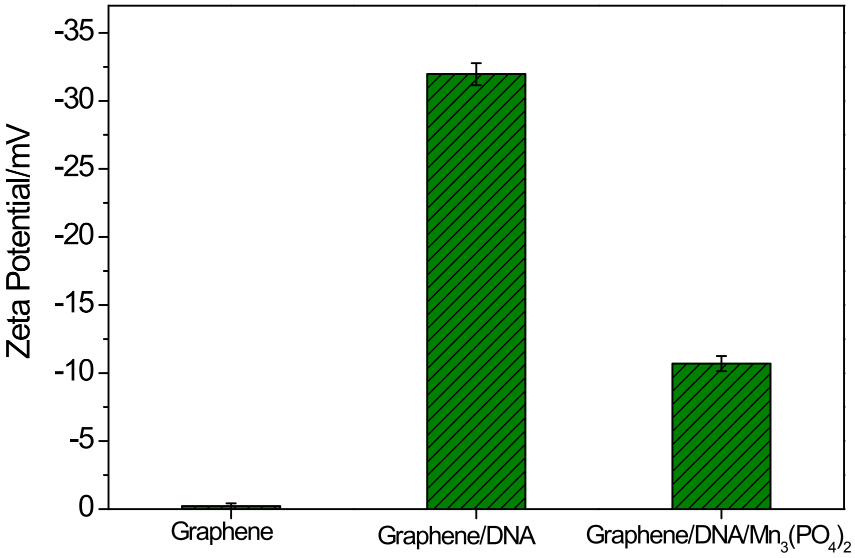

Fig. S2. EDS of the synthesized graphene/DNA/$Mn_3(PO_4)_2$.

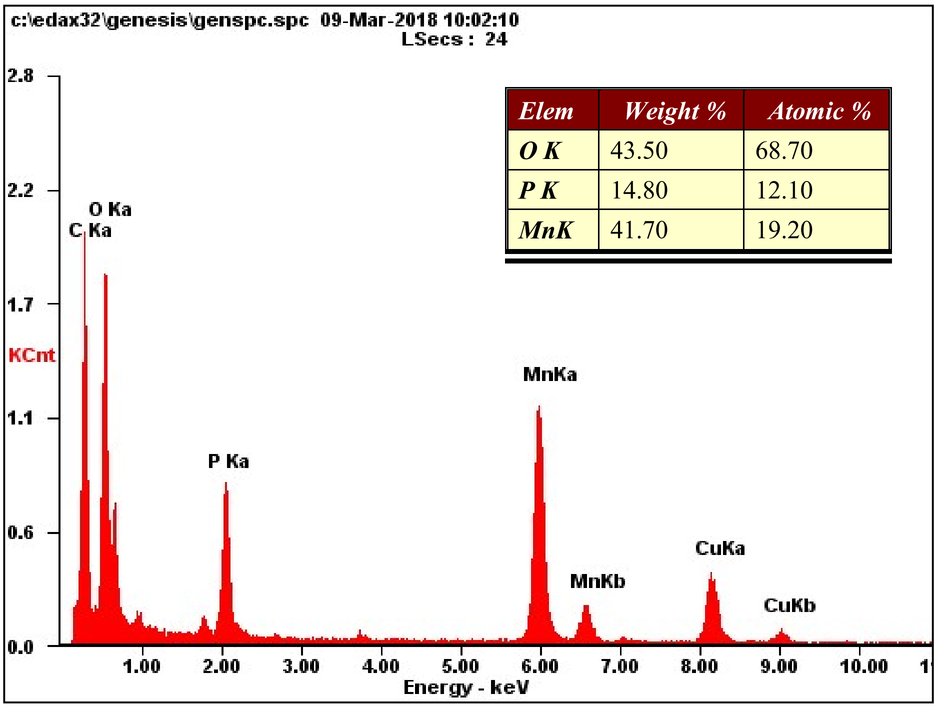

Fig. S3. Voltmmaograms obtained at Graphene/$Mn_3(PO_4)_2$/GCE (A), $Mn_3(PO_4)_2$/DNA/GCE (B), Graphene/DNA/Mn^{2+}/GCE (C) and Graphene/DNA/ $Mn_3(PO_4)_2$/GCE (D) in 0.01M PBS (pH =7.4) and in the presence of 1.0 μM $O_2^{\bullet -}$ and the mixture of $O_2^{\bullet -}$ and SOD (potential scan rate: 50 mV/s).

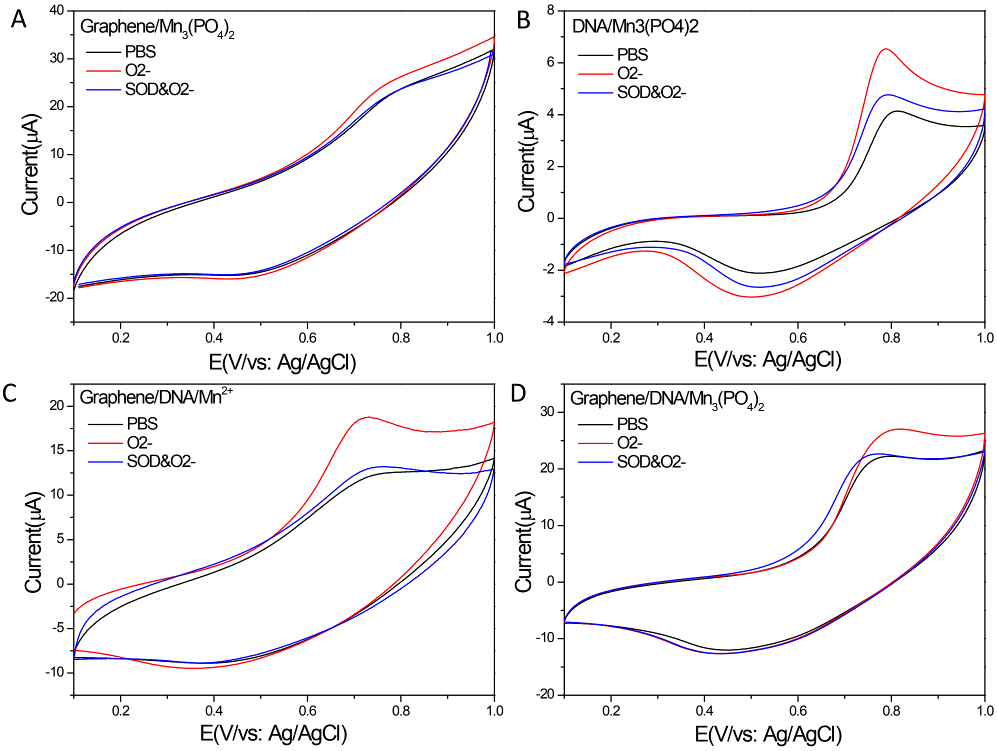

Fig. S4. The stability of the sensor.

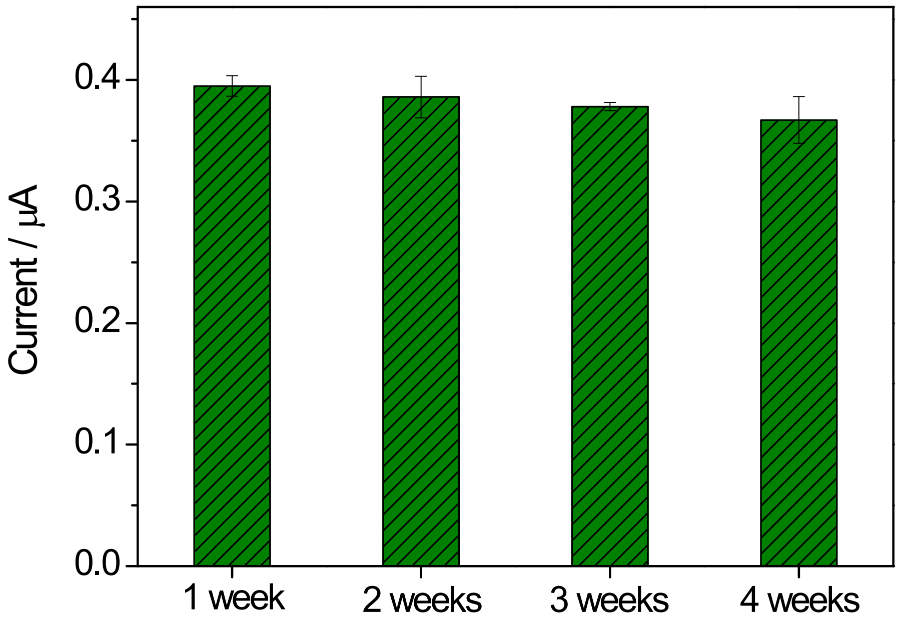

Supplement: RA-008-C7RA12962A-s001 [file RA-008-C7RA12962A-s001.pdf]
